# The Goldilocks paradigm: comparing classical machine learning, large language models, and few-shot learning for drug discovery applications

**DOI:** 10.1038/s42004-024-01220-4

**Published:** 2024-06-12

**Authors:** Scott H. Snyder, Patricia A. Vignaux, Mustafa Kemal Ozalp, Jacob Gerlach, Ana C. Puhl, Thomas R. Lane, John Corbett, Fabio Urbina, Sean Ekins

**Affiliations:** Collaborations Pharmaceuticals, Inc., 840 Main Campus Drive, Lab 3510, Raleigh, NC 27606 USA

**Keywords:** Cheminformatics, Pharmaceutics, Drug discovery and development

## Abstract

Recent advances in machine learning (ML) have led to newer model architectures including transformers (large language models, LLMs) showing state of the art results in text generation and image analysis as well as few-shot learning (FSLC) models which offer predictive power with extremely small datasets. These new architectures may offer promise, yet the ‘no-free lunch’ theorem suggests that no single model algorithm can outperform at all possible tasks. Here, we explore the capabilities of classical (SVR), FSLC, and transformer models (MolBART) over a range of dataset tasks and show a ‘goldilocks zone’ for each model type, in which dataset size and feature distribution (i.e. dataset “diversity”) determines the optimal algorithm strategy. When datasets are small ( < 50 molecules), FSLC tend to outperform both classical ML and transformers. When datasets are small-to-medium sized (50-240 molecules) and diverse, transformers outperform both classical models and few-shot learning. Finally, when datasets are of larger and of sufficient size, classical models then perform the best, suggesting that the optimal model to choose likely depends on the dataset available, its size and diversity. These findings may help to answer the perennial question of which ML algorithm is to be used when faced with a new dataset.

## Abstract (web summary)

Machine learning (ML) is a powerful tool in the field of drug discovery, with the continuous development of new models, however, rational selection of the most appropriate model based on the task remains challenging. Here, the authors explore the capabilities of classical ML algorithms and newer models over a range of dataset tasks and show an optimal zone for each model type, developing a predictive model to aid in the selection of a modeling method based on dataset size and diversity.

## Introduction

The past two decades have seen the meteoric rise of machine learning (ML) as a powerful tool in the field of early drug discovery^1^. Harnessing the ability of ML algorithms to generate predictive models has enabled researchers to virtually screen novel molecules for target-based activity, as well as for predicted absorption, distribution, metabolism, excretion, and toxicology (ADME/Tox) properties that would potentially preclude candidate molecules from use as potential therapeutics^2^. This in silico screening strategy is aimed at decreasing the rounds of in vitro testing required in preclinical stages and improving the overall cost-effectiveness of preclinical drug discovery^1–4^. Some of the most successful ML efforts in drug discovery use quantitative structure-activity relationship (QSAR), quantitative structure-property relationship (QSPR) as well as classification models or other ligand-based strategies to this end^5,6^. Traditionally, such ligand-based modeling involves the training of ML algorithms such as Random Forest (RF), Support Vector Regression (SVR)^7–13^, Naïve Bayes (NB)^14–18^, K nearest neighbor (KNN)^19^, and Deep Neural Nets (DNN)^20–28^ on 2D structural fingerprints (ECFP6, MACCS^29^), physiochemical descriptors (RDKit, Mordred^30^ and many others), or some combination thereof^31^.

These traditional ML approaches often require a significant amount of data before their predictive ability reaches significance, reducing their application to targets or datasets with a substantial number of molecules. Recent prediction methods have utilized Transfer Learning and Multi-task output to take advantage of larger dataset sizes that may exist for biologically related targets, an effort that has been bolstered by the introduction and expansion of state-of-the-art large-language models (LLMs) like the one used by the popular chatbot, ChatGPT^32^. These new modeling architectures are likely rapidly overtaking traditional approaches for performing a variety of cheminformatics analyses. Recurrent neural networks (RNN), and long short-term memory (LSTM) networks^33,34^ have been found to be very useful in a variety of prediction and optimization tasks^35^. More recently, simplified molecular line entry system (SMILES)^36^ strings have been used as input for Sequence-To-Sequence (Seq2Seq) and Transformer models^37^. SMILES represent a natural format for Seq2Seq modeling because the linear encoding of 2D structural information is a good analog for the “word and sentence” structure of Seq2Seq models^38^. However, the performance of Transformer-based architectures in drug discovery has been limited due to the average size of regression and classification datasets; these model architectures require large training sets, encompassing millions of compounds, before state-of-the-art performance emerges^38^. Available structure-activity relationship (SAR) datasets for drug targets, however, often number in the tens of compounds, rendering them unusable for Transformer or even classical ML algorithms. One strategy to navigate this problem is to pre-train Transformer models on large datasets, and then fine-tune them for a single target or endpoint of interest^39,40^. Another approach is to use a modeling technique specifically developed for small datasets, such as few-shot^41^ or zero-shot learning^42^.

As the size of regression and classification datasets can also vary greatly from target to target, and transfer or meta-learning allows for the use of smaller datasets, it can be tempting to apply these newer modeling techniques to all targets in drug discovery. However, few, if any, direct comparisons have been made between classical ML algorithms and these newer models across a wide spectrum of dataset sizes. Here, we evaluate three methods of ligand-based ML modeling at multiple scales of data, including small, medium, and large dataset sizes of different chemical diversities, to find a model selection heuristic for drug discovery. Unsurprisingly, we show that few-shot-learning classification (FSLC) models outperform both transformer (MolBART) and classical ML algorithm support vector classification (SVC) models when trained on small datasets ( < 50 compounds), and SVC models had more predictive power than either the MolBART or FSLC models when the training set exceeded 240 compounds. However, in the “medium” dataset range between 50 and 240 compounds, the advantage of MolBART or SVC modeling becomes dependent on the composition of the dataset, rather than the size. Increasing molecular diversity, quantified by increasing unique Murcko scaffolds in the dataset, favors MolBART modeling over SVC in this middle ground. Because of the “just right” nature of these observations, which consider both size and structural diversity for optimum modeling, we have termed our heuristic the “Goldilocks learning paradigm.” and developed a predictive model to aid in the selection of the modeling method based on inputs of dataset size and diversity. We then tested this paradigm further by modeling five kinases, with vastly different dataset sizes and complexity, that are implicated in the pathology of Alzheimer’s disease (AD). We ultimately show which of the ML approaches performs the best, and in the process, we identify some new inhibitors for MARK1.

## Results

### Transformer models outperform traditional modeling

Our previous use of ML to model enzyme inhibition has relied upon two-dimensional molecular descriptors (e.g., ECFP6) to generate molecular fingerprints, and a suite of traditional ML algorithms to create predominantly classification models^43–45^. More recently we have applied these ML models for regression models for predicting compound activity^46,47^. This approach is completely ligand-based, with the descriptors representing the presence or absence of substructures within the molecule. Each algorithm generates its own model for activity, for which a nested 5-fold cross-validation strategy is performed for hyperparameter optimization and to internally validate the models. The models are then either used to predict the activity of new compounds. To investigate the impact that dataset size has on traditional modeling, we trained SVR models on 2401 datasets of various sizes using a nested 5x cross-validation strategy (see: Large-Language Model Dataset Curation).

**Fig. 1:**
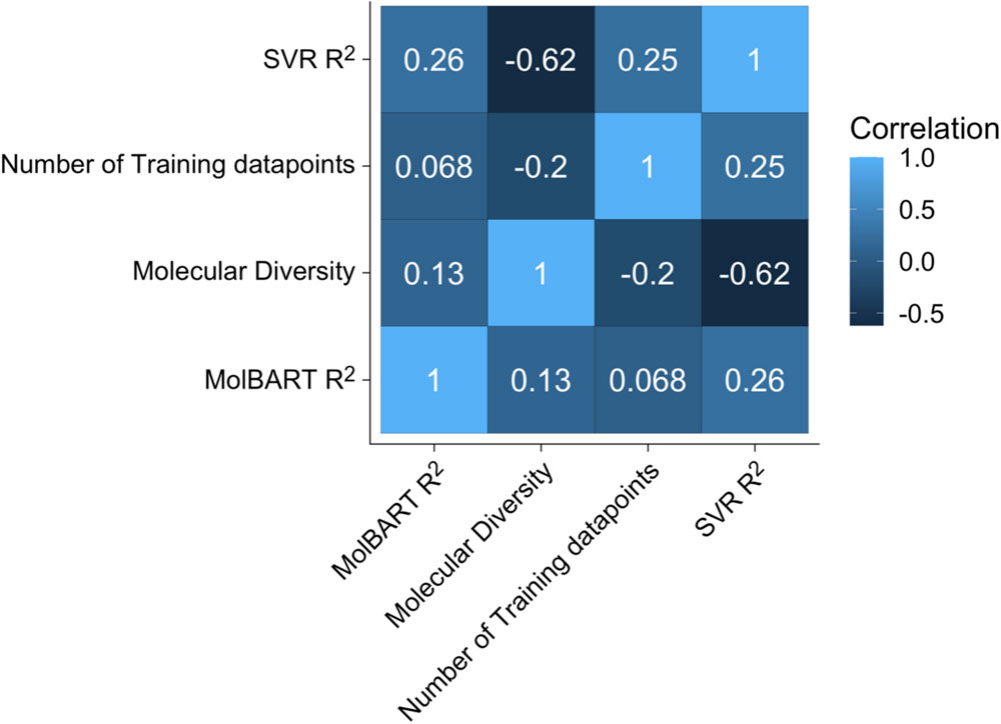
Correlation plots between MolBART *R*^2^, SVR *R*^2^, the molecular diversity of the training set, and the number of molecules in the training dataset. First, each of these metrics was calculated for each individual-target dataset in the original 2401 set from ChEMBL. The correlation was then calculated for each metric.

**Fig. 2:**
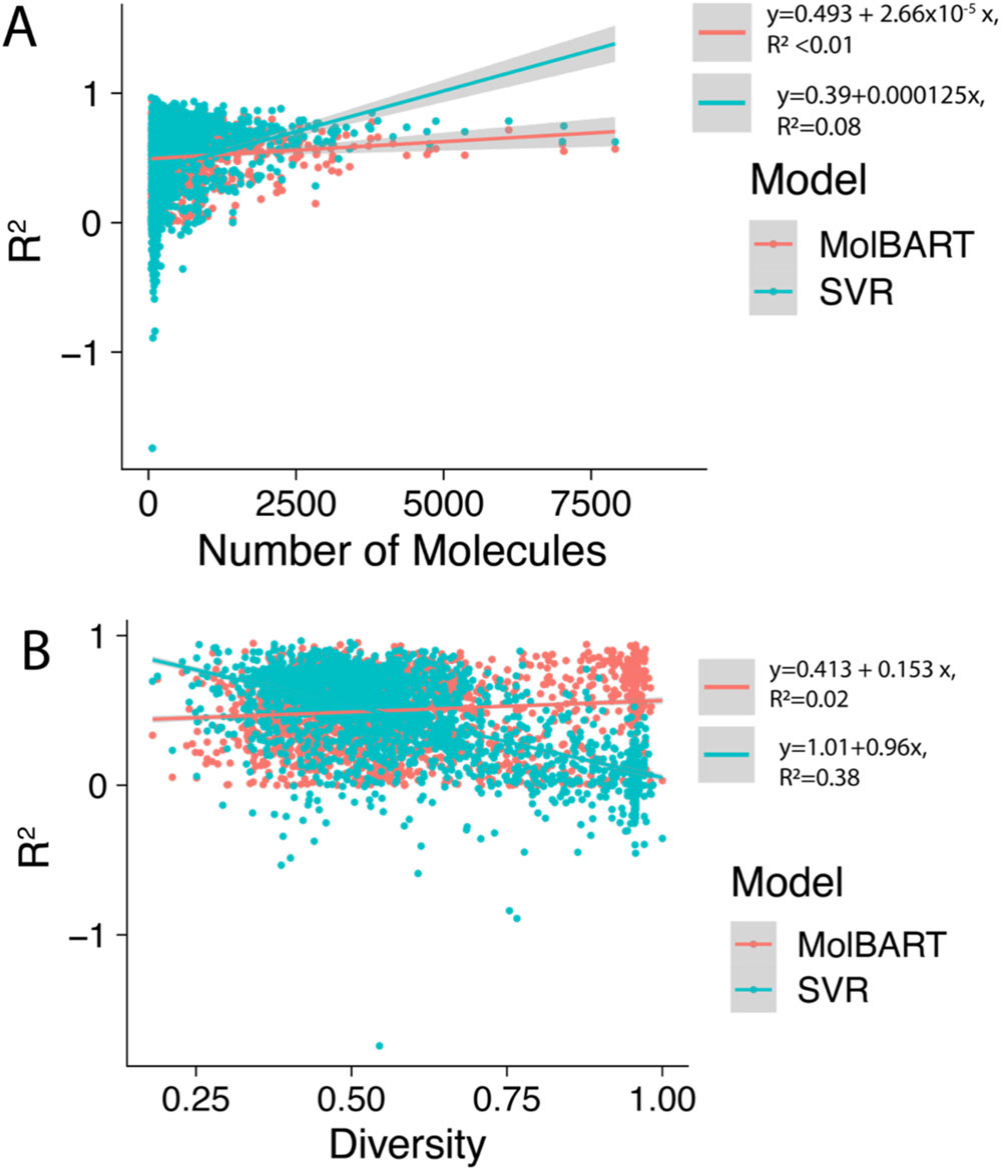
Comparison of MolBART and SVR model *R*^2^ with dataset size and molecule diversity. **A** *R*^2^ vs. the number of molecules for each of the 2401 training datasets from ChEMBL. Each point is a single-target dataset. **B** *R*^2^ vs. the diversity for each of the 2401 training datasets. Each point is a single-target dataset.

**Fig. 3:**
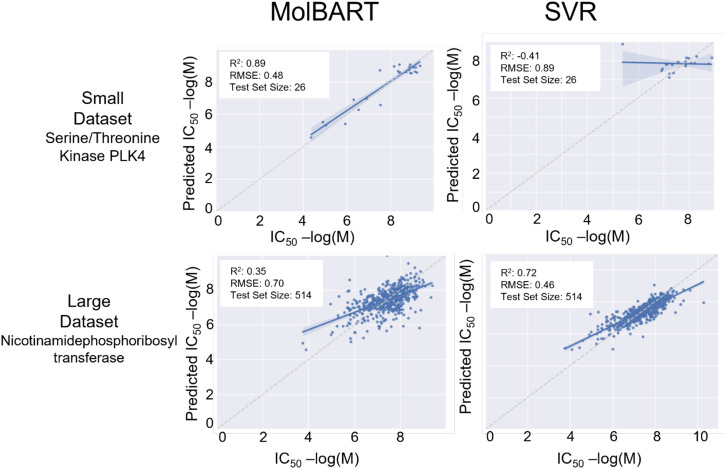
Example true vs. predicted -log(M) values for a small and large dataset for MolBART and SVR. Perfect predictions would appear along the central gray line.

To determine whether large-language transformer models can outperform traditional ML methods, we fine-tuned a pre-trained large-language model called MolBART on the 2401 individual-target datasets from ChEMBL and explored the predictive power of the model in comparison to SVR (Supplementary Data 1). While the comparison to SVR was performed in the original publication, less than 100 datasets were investigated^38^. We first determined whether MolBART would “ignore” small datasets in favor of large datasets, as presumably, MolBART could achieve overall low error rates if it focused on large datasets while ignoring smaller ones. We found that MolBART test prediction statistics were relatively insensitive to the number of endpoints in the target dataset with a correlation coefficient of 0.068 (Fig. 1). This suggests the utility of transfer learning as both large and small datasets can be used for training the model without the unbalanced dataset “overwhelming” the predictive power of smaller dataset endpoints. We next investigated whether the training set size impacted the predictive power of the fine-tuned MolBART model vs. the individual SVR models. The standard cross-validated SVR models have increased *R*^2^ as the number of endpoints for a particular target increases. In contrast, the MolBART *R*^2^ is independent of the number of target endpoints, suggesting the model is taking advantage of transfer learning for low-number datasets (Fig. 2A). Increasing the diversity generally results in a decrease in *R*^2^ for the SVR models but not for MolBART, suggesting the latter can handle more diverse datasets better (Fig. 2B). Comparing two different endpoints for the SVR and MolBART models (Fig. 3) shows the accuracy of predictions with a small dataset size (e.g. opioid receptors, training set size = 80 endpoints, test set size = 20 endpoints) compared to a larger dataset size (Nicotinamide phosphoribosyltransferase, training set size = 2249 endpoints, test set size = 562 endpoints).

**Fig. 4:**
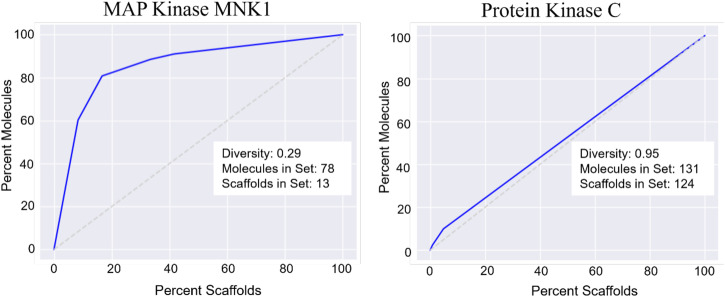
Sample CSFP plot curves of a non-diverse dataset (MAP Kinase MNK1) and a diverse dataset (Protein Kinase C). These images illustrate how the graph changes as diversity decreases. A perfectly diverse dataset with all unique scaffolds would be a straight diagonal line, while a dataset comprised of only one scaffold would encapsulate the entire area of the plot.

The top 10 datasets with the largest differences between MolBART *R*^2^ and SVR *R*^2^ all have <100 training datapoints, suggesting that datasets with the smallest number of endpoints gain the most predictive benefit from transfer learning acquired using pre-trained transformers (Table S1). To identify other factors that might impact the accuracy of prediction, we investigated the structural diversity of each of the training datasets for each of the individual targets. We chose to examine the distribution of molecules within each dataset and the number of unique Murcko scaffolds present^48^. Datasets with a wider array of distinct scaffolds cover more chemical property space and therefore may lead to more generalizable ML models. An established method for identifying the structural diversity of a dataset is to use a Cumulative Scaffold frequency Plot (CSFP)^49^ which compares the percentage of molecules within a dataset that share the same scaffold (Fig. 4 shows how the graph changes as diversity decreases). This is usually plotted as a fraction of molecules, sorted by frequency from the most frequent scaffold to the least frequent scaffold vs. the percentage of unique scaffolds. The scaffolds for each molecule in a dataset were determined using the RDKit Cheminformatics package, with the MurckoScaffoldSmilesFromSmiles function in the rdkit.Chem.Scaffolds.MurckoScaffold module. These plots are similar in structure to ROC curves in that each axis goes from 0 to 100%. A perfectly diverse dataset with all unique scaffolds would be a straight diagonal line, while a dataset comprised of only one scaffold would encapsulate the entire area of the plot. We defined a diversity metric based on the area under the CSFP curve (AUC). We defined our diversity metric as

$${{{{{\rm{div}}}}}}=2(1-{{{{{\rm{AUC}}}}}})$$

Using this metric a perfect diversity score would have a div = 1. If all of the molecules have the same scaffold the dataset would not be diverse at all and so diversity = 0. Figure 1 shows the predictive ability of MolBART independent of target diversity with a correlation of 0.13. However, there is a strong negative correlation between target diversity and the predictive ability of SVR models (Fig. 2B). As the diversity of a target dataset increases there is more structural information within it that needs to be incorporated into the SVR model, making accurate predictions less likely.

**Fig. 5:**
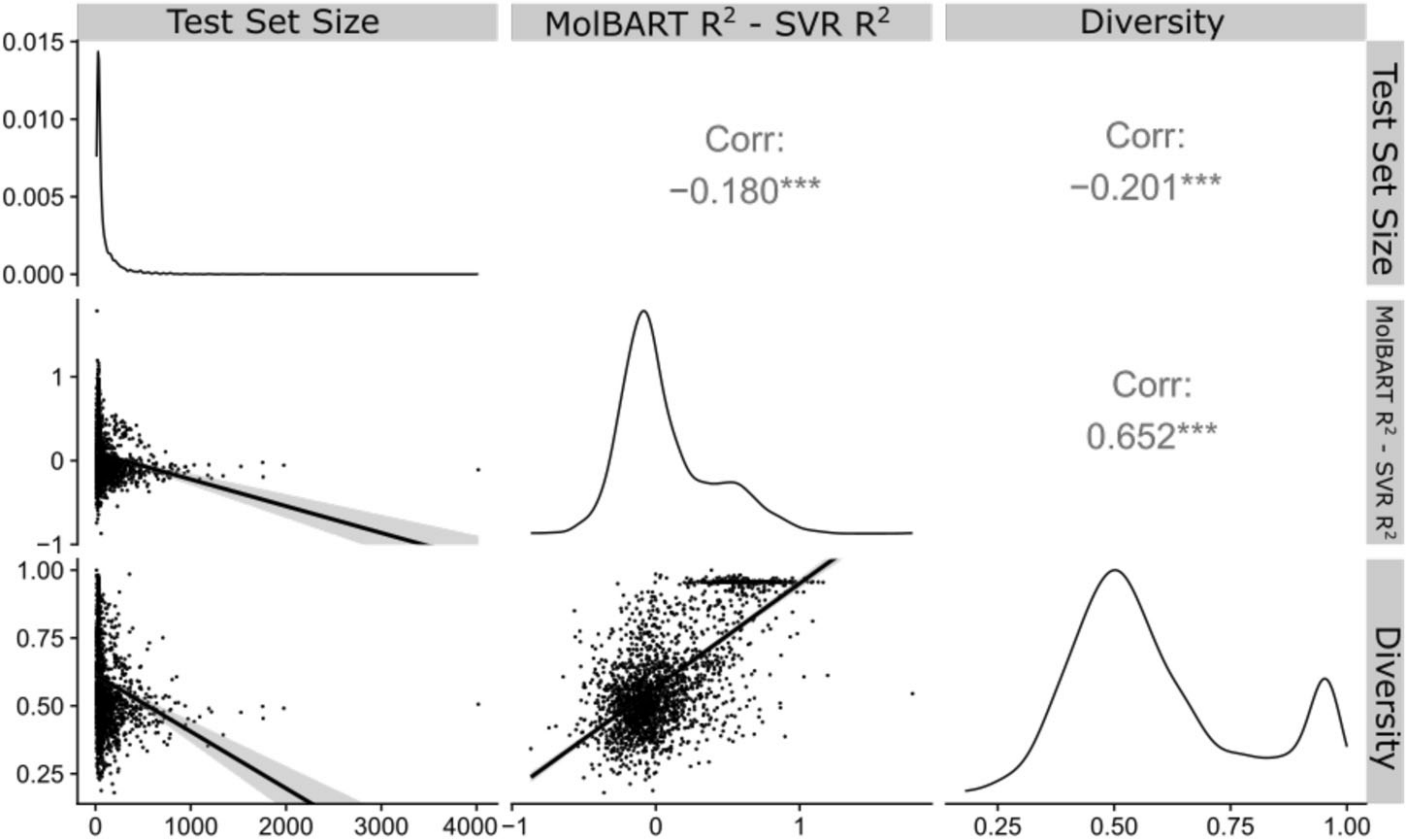
Correlation plots between MolBART *R*^2^–SVR *R*^2^, molecular diversity per training set (diversity), and the number of molecules per training set. Each dot corresponds to a different target dataset. Correlations were significant between each paired feature (*p* < 0.05). Each datapoint represents a single-target dataset.

Exploring the correlation between *R*^2^, dataset diversity, and the number of molecules in a dataset reveals a “sweet spot” in which our pre-trained transformer excels, as well as where traditional ML modeling with ECFP6 tends to outperform the fine-tuned MolBART model. Essentially, when diversity is high and the number of molecules in the training datasets is low (~ <240 datapoints), MolBART however excels at predictive power over SVR. However, as the size of the dataset increases, traditional ML modeling (exemplified by SVR in this case) outperforms MolBART (Fig. 5).

### Few-shot-learning classifiers outcompete large-language models and classical machine learning with extremely low data

While LLMs are performative with high diversity and low dataset numbers, oftentimes predictive modeling is required at even smaller dataset size extremes. Until recently modeling for targets with little data (≤10 known actives and ≤20 total datapoints, herein referred to as micro-data) has been unusable due to limited information in these small datasets. Recently, few-shot and zero-shot learning models have shown state-of-the-art performance in text generation, image classification^50^, and ML model classification predictions for micro-data, paving the way for ML to apply in data-poor situations (for example, these approaches could be used with PROTACS datasets for which there is currently very limited ADME data^51^, or for dark kinases also with limited data^52^). However, the decision point on when to use few-shot-learning models vs. traditional ML modeling or LLMs for modeling training data based on the number of datapoints has not been extensively investigated. Given MolBART’s apparent advantages over the classical learning model at low dataset numbers, we decided to implement and test a prototypical network few-shot-learning classifier (FSLC) to benchmark it (Fig. S1; see few-shot-learning model architecture and training). As FSLC are classification models, we built SVM classification models and fine-tuned the molBART pre-trained model for classification to better compare the model types.

Few-shot-learning models benefit from training on similar tasks to what they will predict, and thus we extracted a subset of kinase-specific target datasets (371 target datasets) from the original 2401 datasets. From these, we eliminated kinase datasets that had less than 20 active and 20 inactive compounds and were left with a total of 95 kinase datasets for training. 64 kinases were used as the training set, with 14 kinases held out for validation and 14 held out for testing. The FSLC is first trained on the 64 separate kinases. To simulate a micro-dataset, 2–20 datapoints are sampled from each training kinase dataset as examples (the support set), and the model uses these datapoints as references for predictions (the test set). Varying sample numbers were drawn to determine the effect of class imbalance and dataset size on the FSLC (Table 1). Once trained, the pre-trained FSLC can predict new molecules similarly for the test set kinases (see methods for training and testing details). Traditional ML models (e.g. SVM) were trained using the same datapoints and dataset size.

**Table 1:** Support set (“training” set) and query set (test set) compositions for different shots during training of FSLC

Support set		Query set	
Actives	Inactives	Actives	Inactives
10	10	10	10
5	10	5	10
1	10	1	10
5	5	5	5
1	5	1	5
1	1	1	1

The number of actives and inactives are given in different amounts and ratios to determine how stable the FSLC classifier is to different training data distributions under extremely small sample sizes.

The pre-trained FSLC could predict correctly even under extremely small support sets of a presented 1 active, 1 inactive dataset (Table 2). In comparison, SVR failed to learn under micro-datasets until at least 5 actives and 5 inactives were presented to the model (Table 3). As the dataset size approached 20 (10 actives and 10 inactives), the classical ML model and the FSCL rapidly converged, suggesting that FSLC are powerful below the 20 datapoint mark, but lose comparative power once the datasets are large enough.

**Table 2:** Summary of the classification metrics for FSLC trained with graph descriptors for 14 validation tasks

Shots	ROCAUC	AVG. PRECISION	F1	CK	MCC
10-10	**0.684** ± **0.070**	0.694 ± 0.174	**0.620** ± **0.106**	**0.237** ± **0.112**	**0.248** ± **0.111**
5-10	0.652 ± 0.088	0.676 ± 0.164	0.599 ± 0.096	0.200 ± 0.136	0.213 ± 0.139
1-10	0.660 ± 0.072	**0.707** ± **0.130**	0.546 ± 0.113	0.181 ± 0.113	0.197 ± 0.120
5-5	0.650 ± 0.078	0.663 ± 0.141	0.563 ± 0.129	0.175 ± 0.106	0.188 ± 0.110
1-5	0.658 ± 0.073	0.690 ± 0.153	0.581 ± 0.113	0.210 ± 0.122	0.221 ± 0.123
1-1	0.640 ± 0.109	0.662 ± 0.157	0.561 ± 0.123	0.165 ± 0.112	0.180 ± 0.124

Values for each metric are mean and standard deviation. The highest scores are bolded.

**Table 3:** Summary of the classification metrics for the SVC models trained with ECFP6 descriptors for 14 validation tasks

Shots	ROCAUC	AVG. PRECISION	F1	CK	MCC
10-10	**0.645** ± **0.079**	**0.630** ± **0.188**	**0.617** ± **0.129**	**0.261** ± **0.155**	**0.280** ± **0.156**
5-10	0.564 ± 0.070	0.610 ± 0.171	0.250 ± 0.163	0.113 ± 0.150	0.176 ± 0.145
1-10	0.503 ± 0.004	0.574 ± 0.169	0.012 ± 0.016	0.005 ± 0.008	0.016 ± 0.014
5-5	0.604 ± 0.078	0.607 ± 0.174	0.566 ± 0.124	0.191 ± 0.155	0.212 ± 0.155
1-5	0.503 ± 0.003	0.055 ± 0.165	0.018 ± 0.016	0.007 ± 0.007	0.022 ± 0.011
1-1	0.531 ± 0.035	0.561 ± 0.159	0.442 ± 0.098	0.059 ± 0.070	0.081 ± 0.078

Values for each metric are mean and standard deviation. The highest scores are bolded.

Our results suggest that the different learning modalities have strengths and weaknesses at different levels of data information. To determine if this relationship is computationally predictable, we trained an ML model to predict which approach (MolBART, SVM, or FSLC) is likely to have the highest predictive power using Fast Interpretable Greedy-Tree Sums (FIGS)^53^. FIGS is a generalized classification and regression tree (CART) model, which creates highly interpretable decision trees from decision leaves in the model. We split the 95 kinase datasets (described above) into a training and test set. We set the hyperparameter number of trees = 3 and used only dataset size and molecular diversity as the input, and the output was a multi-class decision (0,1,2 for molBART, SVM, and FSLC respectively). Using the holdout test set of datasets that were not seen during training, the FIGS classifier was able to predict the correct winning ML model type (Table 4, ROC 0.74). The decision tree produced by the FIGS classifier (Fig. S2) gives a heuristic decision tree, suggesting that relative model performance can be predicted based on dataset size and diversity alone.

**Table 4:** FIGS classifier statistics when predicting which machine-learning model (MolBART, SVM, FSLC) will perform best when using dataset size and dataset diversity as input

ROC	Precision	Recall	Accuracy	Specificity	MCC
0.74	0.77	0.63	0.75	0.84	0.49

From these results, we propose a simple heuristic for model selection based on the dataset size: For datasets <50 molecules, FSLCs dominate. From 50-240 molecules and particularly for diverse datasets, LLMs such as MolBART outperform other modeling types. Finally, as dataset size increases>240 molecules, traditional ML algorithms such as SVR models with ECFP-feature inputs are recommended.

### The Goldilocks zone for model selection: discovery of novel kinase inhibitors for Alzheimer’s disease

To compare these ML model types and show the application of where each of them excels, respectively, we built and applied ML models to discover new kinase inhibitors for AD. The mechanisms underlying the AD pathophysiology are still unclear. Aggregation of tau and amyloid beta (Aβ) proteins as well as decreased acetylcholine are the focus of many studies^54^. Neurofibrillary tangles (NFTs) are one of two hallmark plaques in AD^55–57^ and are comprised of a hyperphosphorylated version of the microtubule-stabilizing protein tau (Ptau)^58^. Ptau dissociates from the microtubule, migrating away from the axon, and forming insoluble paired helical filaments in the cytoplasm of the soma. This leads to destabilization and loss of the cytoskeletal microtubule, part of a cascade of events that leads to neuronal death^58^. Three major classes of kinases can phosphorylate tau^59^: proline-directed kinases like glycogen synthase kinase 3 beta (GSK-3β) or cyclin-dependent kinase 5 (CDK5)^60,61^, non-proline-directed kinases such as tau-tubulin kinases (TTBK)^62^ or microtubule affinity regulated kinases (MARK)^63^, and tyrosine kinases such as Fyn^64^ or Abl kinases^65^. Using IC_50_ data from the ChEMBL database and ECFP6 Fingerprints with 1024 bits, we built ML classification and regression models for GSK3β, ABL1, FYN, CDK5, and MARK1. The classification models for FYN, CDK5, and MARK1 were built with activity thresholds of 1 µM, meaning compounds with IC_50_ values lower than 1 µM are classified as active (Table 5 and Table S2). The kinase inhibitor datasets spanned several orders of magnitude in size, from 18-2969 datapoints and differing molecular diversity, making them an excellent dataset to investigate the Goldilocks zone model selection process. The datasets for both GSK3β and ABL1 contained sufficient entries of low-nanomolar inhibitors that we could build good classification models (according to the cross-validation statistics generated) based on the lower activity threshold of 100 nM (Table 5 and Table S2). The MARK1 dataset was by far the smallest dataset and therefore has the least predictive power for classical ML algorithms, which is reflected in the internal cross-validation scores for the classification models (Table S2). The fine-tuned MolBART model was further trained on each of these datasets, following the same training procedure as performed in the original fine-tuned training of the model.

**Table 5:** Training and test sets for classification models from publicly available databases

Target	Number of compounds in the training set (ChEMBL)	Activity threshold of training set	Number of active/inactive compounds in the training set	Dataset diversity	Number of compounds in external test set (BindingDB)
GSK3β	2969	100 nM	854/2115	0.52	319
ABL1	1791	100 nM	856/935	0.52	440
FYN	1070	1 µM	98/972	0.74	656
CDK5	309	1 µM	103/206	0.61	310
MARK1	18	1 µM	5/13	-	-

**Fig. 6:**
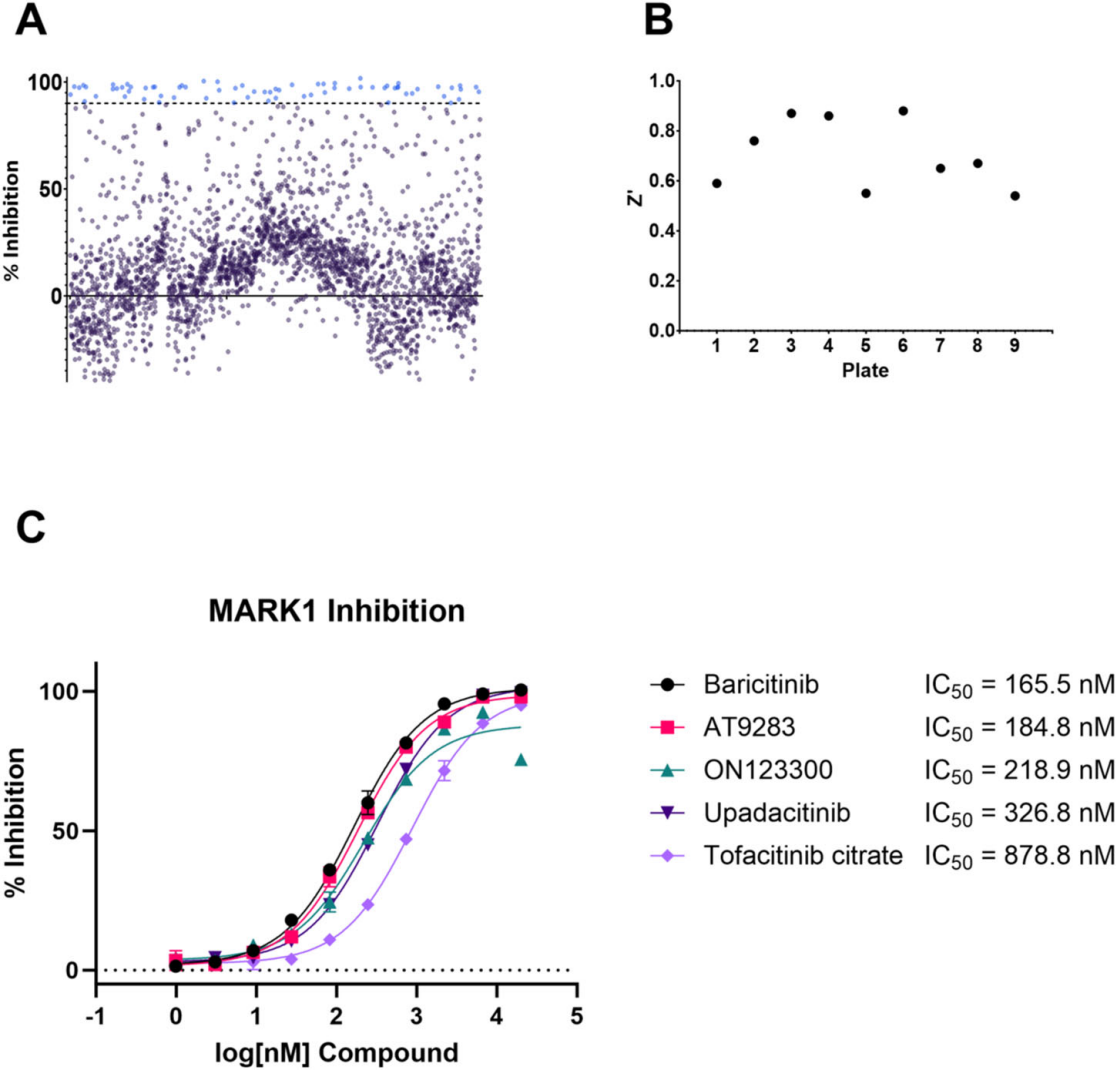
Developing a Test Set for MARK1 Inhibition. **A** The MedChemExpress FDA-Approved and Pharmacopeial Drug Library (HY-L066) was screened for MARK1 inhibition using the Promega ADP-Glo Kinase Assay at a concentration of 385 µM. Compounds that exhibited >90% inhibition are shown in light blue. **B** The Z-factor for each of the nine plates used in the screen. **C** IC_50_ value determination for five novel MARK1 inhibitors using Z’-LYTE assay. Non-linear regression analysis (3-parameters) was performed in GraphPad Prism. Error bars are standard deviation.

The differences in the predictive power of the three model types, track with what we have seen from our own internal inhibition testing and model predictions for these four kinases and from the earlier comparative analysis (Table S3) with the FYN and CDK5 models performing less well. MARK1 presents a unique challenge, as it has sparse data and no external test set (Table 5). Because we did not have an external test set for our MARK1 model, and no measure of confidence for our classical ML model predictions, we performed a high-throughput screen of FDA-Approved compounds for MARK1 activity to create a prospective test set (Fig. 6A, B). We virtually screened the FDA-approved library with our classical models, fine-tuned MolBART model, and our pre-trained Kinase FSLC model to determine model performance with the sparse MARK1 dataset. The MedChemExpress FDA-Approved and Pharmacopeial Drug Library (HY-L066) was experimentally screened for MARK1 inhibition using the Promega ADP-Glo Kinase Assay. We selected a subset of hits from our high-throughput screen, along with a few compounds from our internal library, to be tested for IC_50_ value determination (Fig. 6C), and then used those results as a test set for our MARK1 models.

**Fig. 7:**
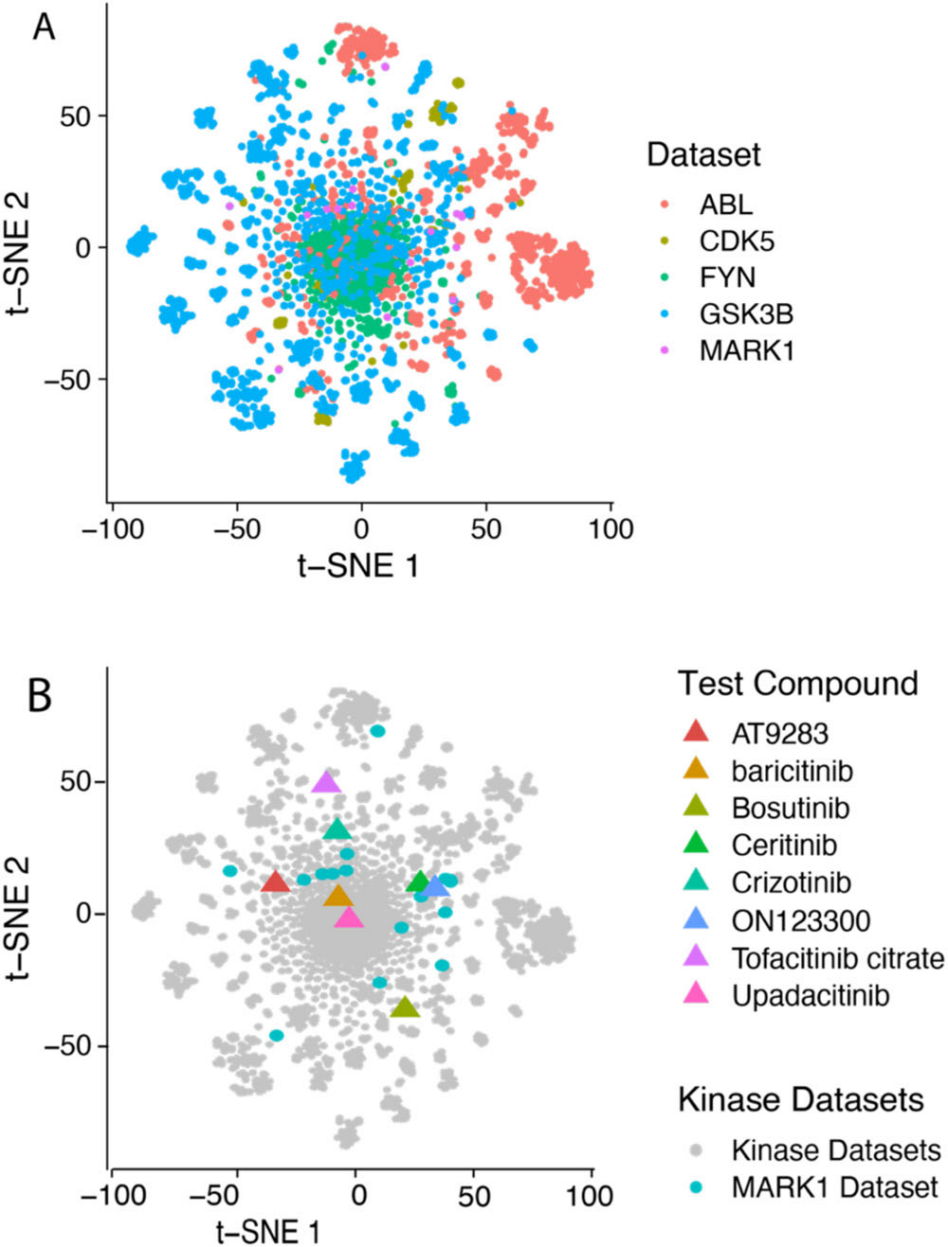
t-SNE plots of the MACCS key fingerprints of the kinase datasets and the discovered MARK1 inhibitors. **A** Chemical space overlap of the kinase datasets. **B** Chemical space overlap of the discovered MARK1 inhibitors vs. the MARK1 dataset and the remaining kinase datasets.

Out of the 13 tested compounds, 5 (baricitinib, AT9283, ON123300, upadacitinib, and tofacitinib citrate) were true novel MARK1 active inhibitors that to our knowledge had not been described previously with this activity (Fig. 6C and Table S4). Baricitinib is a JAK1 and JAK2 inhibitor^66^. AT9283 is an aurora kinase and JAK2 inhibitor^67,68^. ON123300 is a CDK4, PI3K/AKT/mTOR inhibitor^69–71^. Upadacitinib is a JAK-1 inhibitor^72^. Tofacitinib is a JAK1 and JAK3 inhibitor^73^. We have also compared the maximal Tanimoto similarity (using MACCS key fingerprints) of the compounds to the MARK1 training set, with the closest being 0.79 (tofacitinib), while the similarity of the majority of the other hits ranged from 0.56-0.75, suggesting some structural diversity compared to the training dataset. We next visualized these molecules using t-SNE (Fig. 7 and Table S5). Each of the kinase datasets reveals distinct coverage of chemical space (Fig. 7A). When we compare the MARK1 hits discovered, they fall in various regions of chemical space that do not overlap with the known MARK1 training data, indicating the machine-learning model can find unique chemical structures.

Although the classical ML model SVR performed the best followed by MolBART and the FSLC on the external test sets, the inverse performance was seen for the MARK1 prospective test set (Table 6). The SVR model predicted no actives for MARK1. MolBART, even though it was only trained on a test set of 18 compounds, was capable of discovering 3 of the 5 novel inhibitors. This tracks with the notion that pre-trained LLMs can be used to improve predictive power in sparse data situations. FSLC, despite performing worse overall when trained on large datasets, excelled at discovering novel MARK1 inhibitors, finding all 5 inhibitors with high precision (Table 6).

**Table 6:** Truth table of the predictions of SVC, MolBART, and FSLC on the MARK1 prospective test set

Algorithm	TP	TN	FP	FN	Precision
SVC	0	8	0	5	0
MolBART	3	8	0	2	0.6
FSLC	5	7	1	1	1

## Discussion

There have been several large-scale comparisons of ML models using on the order 1000 s datasets^74–78^. We have previously described using over 5000 datasets from ChEMBL with the ECFP6 fingerprint descriptor and provided a comparison of various ML algorithms. In this case, the model performance was assessed using five-fold cross-validation metrics as well as F1 score, Cohen’s kappa, and Matthews correlation coefficient. We created ranked normalized scores for the metrics for all methods and showed that they appeared comparable while the Bayesian algorithm and support vector classification were the best performing^43^. Other very large-scale evaluations include that of Novartis Institute for Biomedical Research which used 8558 proprietary Novartis assays to generate Random Forest Regressor models for their datasets^79^.

Recent advances in ML have shown that transformer-based large-language models scale with the size of the data and number of parameters^80^. While QSAR modeling has been performed with large datasets, the total number of endpoints and thus number of datapoints for training a single model have remained relatively small in comparison to the available data. In this study, we first evaluated the effect of dataset size using 2401 datasets on SVR models and compared performance with fine-tuning a pre-trained MolBART model. We found that the *R*^2^ improved with dataset size for SVR but there was no effect of dataset size on MolBART. As dataset diversity increases the SVR model *R*^2^ decreases whereas with the MolBART model *R*^2^ was independent of diversity. This suggests that the pre-training of the MolBART model allowed it to capture relevant information for all QSAR tasks, giving it a higher predictive power over the SVR models on smaller datasets, which must learn all the relevant QSAR information from the individual datasets. As the dataset size increases, however, SVR models become more specialized in comparison to the multi-endpoint predicting MolBART model, allowing it to capture more nuanced information for each single dataset. Interestingly, the diversity of the dataset correlated negatively with the predictive power of SVR models, suggesting that similar feature representation may play a stronger role in the support vectors generated for the model decision boundary.

Often when we embark on drug discovery projects for new targets there is generally little if any data available to build ML models. In these cases, we traditionally would use approaches like pharmacophores to perform virtual screening based on molecular shape for a few hits^5,6^. In addition, when we are asked by our colleagues or collaborators to build ML models, we are also often queried on how many molecules are required in order to build a ‘useful model?’. We have now explored an ML approach that handles extremely low data called FSCL^50^. Initially, we used a subset of human kinases and simulated small datasets, and directly compared how this FSLC method performed relative to SVR. We found that FSCL performed well with small datasets (5 actives and 5 inactives) and as these numbers doubled its advantages decreased in comparison to both MolBART and SVR models.

Our results suggest that each model type (classical SVM, pre-trained LLM, and the FSCL) occupies a niche in which its predictive power excels over alternatives and that a Goldilocks zone exists for different ML model types. When the dataset is small (<50 molecules), FSLC performs best, capable of generalizing class-features from a small set of representations. When the datasets are diverse with a range of 50-240 molecules, pre-trained LLMs such as MolBART tend to outperform other ML approaches, leveraging the transfer learning from pre-training to outperform SVM while learning more from the slightly larger dataset size compared to FSLC. Finally, as the molecule dataset size increases past 240 datapoints, then classical ML methods such as SVR will dominate. The ability to predict which model algorithm is most likely to outperform the others using just the dataset size and dataset diversity as input suggests this relationship generalizes across most target datasets, giving us a heuristic with which to decide “how should I model my data?”.

We further demonstrate the relevance of these findings using the identification of inhibitors for GSK3β, ABL1, FYN, CDK5, and MARK1 for AD. Using the extreme of a small training dataset of 13 compounds and FSLC, We identified 5 new inhibitors of the kinase MARK1 (most of which are also known as JAK inhibitors) which regulates microtubule dynamics in neurons^81^ and phosphorylates tau which results in cellular transport inhibition, impacts postsynaptic molecular makeup and can induce either spine enlargement or tau toxicity as well as spine decay-depending on expression levels and duration^82^. Targeting tau phosphorylation is a valid target for AD that can form neurofibrillary tangles inside neurons and lead to neuronal death^58^. The MARK1 dataset provided an example to compare the FSLC, MolBART, and classical ML approaches. Our results demonstrated that for a small dataset, FSLC performed the best at identifying the active molecules, outperforming MolBART and classical ML methods. MolBART was able to predict 3 of the 5 inhibitors while SVM predicted 0 inhibitors correctly, demonstrating the Goldilocks zone application.

Important limitations of this study to consider are that we have focused on kinase inhibitors which: 1. are all very similar; 2. there is more data than for other targets; and 3. there is generally a high degree of similarity between targets. To counter this, we would add that in the case of MARK1 there was limited data available, and it was an accessible assay which we could in turn generate new data for. We would suggest that future work could explore our approach with additional targets outside of kinases. For example, we have previously used ML to perform virtual screening to identify new ligands for various GPCRs^83,84^. In addition, we did not explore other molecular descriptors or take into account any of the target 3D structures in this study. We solely focused on ChEMBL and BindingDB as sources of public data for modeling and there may be other published datasets we could include to increase our dataset size in the future. It is also important to remember we are curating data from many publications so there will be considerable variability and experimental error for some assays which we also need to consider for each dataset as well as dataset composition bias. The approach we have taken could also be used to explore and compare many other ML approaches beyond the few described here.

While there is currently considerable interest in methods such as LLMs, they may not be a panacea for our ML modeling of drug discovery relevant datasets just yet for the reasons mentioned. While one advantage of them is that they enable transfer learning across datasets and the construction of a single model that can output predictions for all the targets. We now describe where this algorithm may be the most useful, where the datasets may be midsize and diverse. It will be important to continue to explore whether this Goldilocks paradigm holds up as we look at larger datasets in the future. In the meantime, we have provided some recommendations that may assist others in their selection of which ML methods may be appropriate for datasets of various sizes and diversity.

## Experimental section

### General dataset curation

Publicly available datasets for Fyn, MARK1, Abl, and GSK3β, were downloaded from ChEMBL and BindingDB^85,86^, then standardized with our proprietary “E-Clean” software to remove salts, and neutralize charges, and assign InChIKeys and canonical SMILES using open-source RDkit functions. Continuous activity values were converted to −log[M], and duplicate molecules were averaged based on InChIKey. The Assay Central software^43^ further standardizes datasets using Indigo Toolkit 7 to, dearomatize, standardize, and reposition stereo bonds, standardize or flag erroneous charges, flag erroneous valences, remove isotopes, remove dative and hydrogen bonds, flag multicomponent chemicals, and remove any remaining duplicates.

### Large-language model dataset curation

Over 5000 datasets were curated from ChEMBL^86^ as described previously^43^, and original activity thresholds were retained. Datasets with fewer than 20 total datapoints were removed, resulting in 2401 individual-target endpoint datasets for *K*_i_, IC_50_, or EC_50_ activity. The endpoints for the aggregated dataset were randomly split into training/validation/test sets by following a 70/5/25 split. All datasets were then subjected to the “cleaning” procedures described above.

### Few-shot-learning dataset curation

Datasets for 371 individual kinases were downloaded from ChEMBL. Datasets with fewer than 20 datapoints for either active or inactive compounds were removed, leaving only 95 kinase datasets. These were binarized on a threshold of 100 nM (IC_50_, ≤100 nM or −log[M] ≥ 7), then randomly split into train/validation/test sets by following a 70/15/15 split. All datasets were then subjected to the “cleaning” procedures described above.

### Assay central model building

Our proprietary AC software^43^ uses multiple classic ML algorithms that are integrated into our web-based software to build classification models using the following algorithms: deep learning, adaboost classifier, Bernoulli Naïve Bayes, *K* nearest neighbors (kNN) classifier, logistic regression, random forest classifier, Support vector classification (SVC) and XG boost (XGB) classifier. For model building of continuous data, we have implemented multiple regression algorithms which include adaboost regression, Bayesian regression, elastic net regression, kNN regression, random forest regression, support vector machine regression, and XGboost regression. In all cases, nested 5-fold cross-validation was performed except for deep learning for which we removed 20% of the training set, in a stratified manner for the classification models, and these were used as external test sets for models trained on the remainder of the data.

### Large-language model architecture and training

To explore the effect of “tuning-dataset” size for a pre-trained LLM transformer model, we fine-tuned the base Chemformer pre-trained model provided by Irwin et al.^38^ on progressively larger sets of data. The Large-Language Model is a molecule pre-trained Bidirectional Auto-regressive Transformer (molBART). The BART architecture uses both encoder and decoder layers of the Transformer models, allowing the model to learn contextual molecule encodings (using the encoder) while the auto-regressive decoder module learns molecular structure. After pre-training molBART, the bidirectional encoder can be fine-tuned for downstream tasks such as property predictions (e.g., IC_50_ prediction of molecules against a specific target). The fine-tuning of the pre-trained model was performed using PyTorch using the Lightning^87^ framework for 150 epochs and each of the 2401 endpoint datasets was used in the fine-tuning.

### Few-shot-learning model architecture and training

Few-shot-learning classification (FSLC) models were originally introduced for multi-class image and text classification^50^. However, our application is different as we only have two *classes* for the majority of the *tasks* (active or inactive). The few-shot-learning network was trained on a select number of tasks (e.g. kinase dataset), which have sufficient data and tested on the remaining kinases. *Shot* is the number of molecules sampled per class, and added to the support set during each round of training, also known as *episode*.

FSLC consists of three modules the first two take in molecular descriptors and create embeddings for the molecules. The last module creates “prototypes” for each class by taking the average of molecular features and making predictions, similar to the previous one/few-shot-learning models^42,88,89^. FSLC uses episodic learning to match training and testing conditions. In each episode, the training algorithm samples two batches of labeled, non-overlapping datapoints from each class in each task. One batch is used as the support set, *S, to* create prototypes. The other batch is the query set, *B*, which is used to calculate loss and update the network’s parameters. *g*′ and *f*^′^, and *g* and *f* are the same embedding modules. During testing, support and query sets are sampled for each unseen kinase. Embeddings are created for each new compound, and binary labels are predicted based on the distance between the query compounds and the class prototypes. We used the same FSLC architecture described in Vella and Ebejer^42^. The first module, *g*′, is either a fully connected feed-forward network (FNN) or a graph convolutional network (GCN) depending on the chemical descriptors used. When we trained an FSLC with ECFP chemical descriptors generated with rdkit (2048 bit vectors and radius = 5), we used an FNN architecture. We used GCN when we used the graph convolution-learned embeddings^90^. The second module, *g* is a long short-term memory (LSTM) network with iterative refinement (IterRefLSTM). The details of IterRefLSTM can be found in Altae-Tran et al.^89^. Briefly, the IterRefLSTM uses a context-based attention mechanism to refine the initial embeddings iteratively and simultaneously for the support and query embeddings. The third module is a Prototypical Network (PN) which creates class prototypes and predicts the label of unseen datapoints based on the Euclidean distance. A more detailed explanation can be found in the original Prototypical Networks paper by Snell, Swersky, and Zemel^50^. We trained a number of FSLCs using 1 and 10 shots. FSLCs were trained until the loss was < 1*e*^*−*6^. We used the negative log-likelihood loss and Adam optimizer^91^ to optimize the model parameters. We used the AUC of the PRC to evaluate the model’s general performance similar to Altae-Tran et al*.*^89^ and Vella and Ebejer^42^. We tested the models by randomly sampling each test task 1000 times. The average statistics scores were calculated for each task and for all models.

### In vitro screening

#### Chemical library

The FDA-Approved & Pharmacopeial Drug Library (MedChemExpress, Monmouth Junction, NJ) contains 2743 compounds that were approved by institutions (FDA, PDMA, EMA, or NMPA), or contained in pharmacopeia (JP, BP, EP, or USP). The portion of the library supplied at 10 mM in DMSO (2637 compounds) was screened for MARK1 inhibition. 200 nL of each compound was dispensed into a 384-well assay plate (Perkin Elmer 6008280) using a Mosquito HTS (TTP Labtech Melbourn, UK), and stored at −80 °C until use.

#### Experimental procedures

Compounds were evaluated for MARK1 inhibition using the ADP-Glo™ Assay with the MARK1 Kinase Enzyme System from Promega. Reactions were performed using 10 µM ATP, 0.2 µg/µL CHKtide substrate, 50 µM DTT, and 5 ng MARK1 enzyme, final concentration. All but plate HYCPK12959 contained 0.01% Triton-x 100. The final concentration of the compounds was 385 µM in 3.8% DMSO. Compounds were pre-incubated with enzyme for 10 min at room temperature before the addition of ATP and substrate. The kinase reaction was incubated for 60 min at room temperature, followed by a 40-min incubation with ADP-Glo reagent, then a 30-min incubation with Kinase Detection Reagent. The resulting luminescent signal was read on a SpectraMax iD5 (Molecular Devices, San Jose, CA), using an integration time of 1000 ms.

### Data analysis

Statistical analysis was performed in Excel and GraphPad Prism 9.5.1. Z’ factor was calculated using the formula ${Z}^{{\prime} }=1-(3x({\sigma }_{p}+{\sigma }_{n}))/|{\mu }_{p}-{\mu }_{n}|$.

### Supplementary information

Supplementary Information

Description of Additional Supplementary Files

Supplemental Data 1.

## Data Availability

All relevant data are available from the authors upon written request.
