# Supplementary material for: The Goldilocks paradigm: comparing classical machine learning, large language models, and few-shot learning for drug discovery applications
Source: Commun Chem. 2024 Jun 12;7:134. doi: 10.1038/s42004-024-01220-4 (PMC11169557; doi:10.1038/s42004-024-01220-4)
Supplement: Supplementary file 1 — Supplementary Information [file 42004_2024_1220_MOESM1_ESM.pdf]

Supplementary Information

The Goldilocks Paradigm: Comparing Classical Machine Learning, Large Language Models, and Few-Shot Learning for Drug Discovery Applications

Scott Snyder, Patricia A. Vignaux, Mustafa Kemal Ozalp, Jacob Gerlach, Ana C. Puhl, Thomas R. Lane, John Corbett, Fabio Urbina* and Sean Ekins*

Collaborations Pharmaceuticals, Inc., 840 Main Campus Drive, Lab 3510, Raleigh, NC 27606, USA.

Supplementary Figures

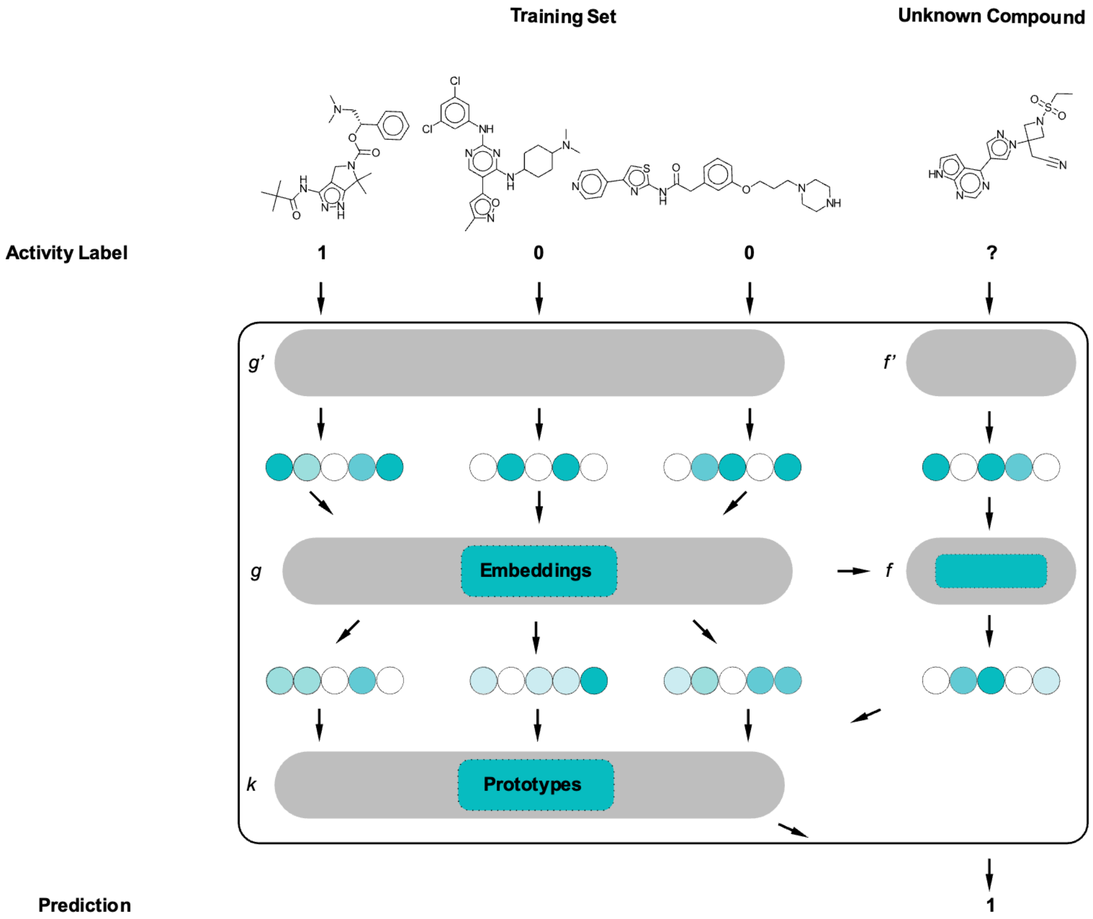

Figure S1. Schematic of few-shot learning network based on Vella and Ebejer. *g′* and *f′* represents the same network architecture for creating initial embeddings. When we used ECFP chemical descriptors, they were fully connected feed-forward neural networks. Otherwise, they were graph convolutional neural networks which took molecular graphs as the input. *k* is the prototypical network. Compounds listed are Pubchem IDs (PID).

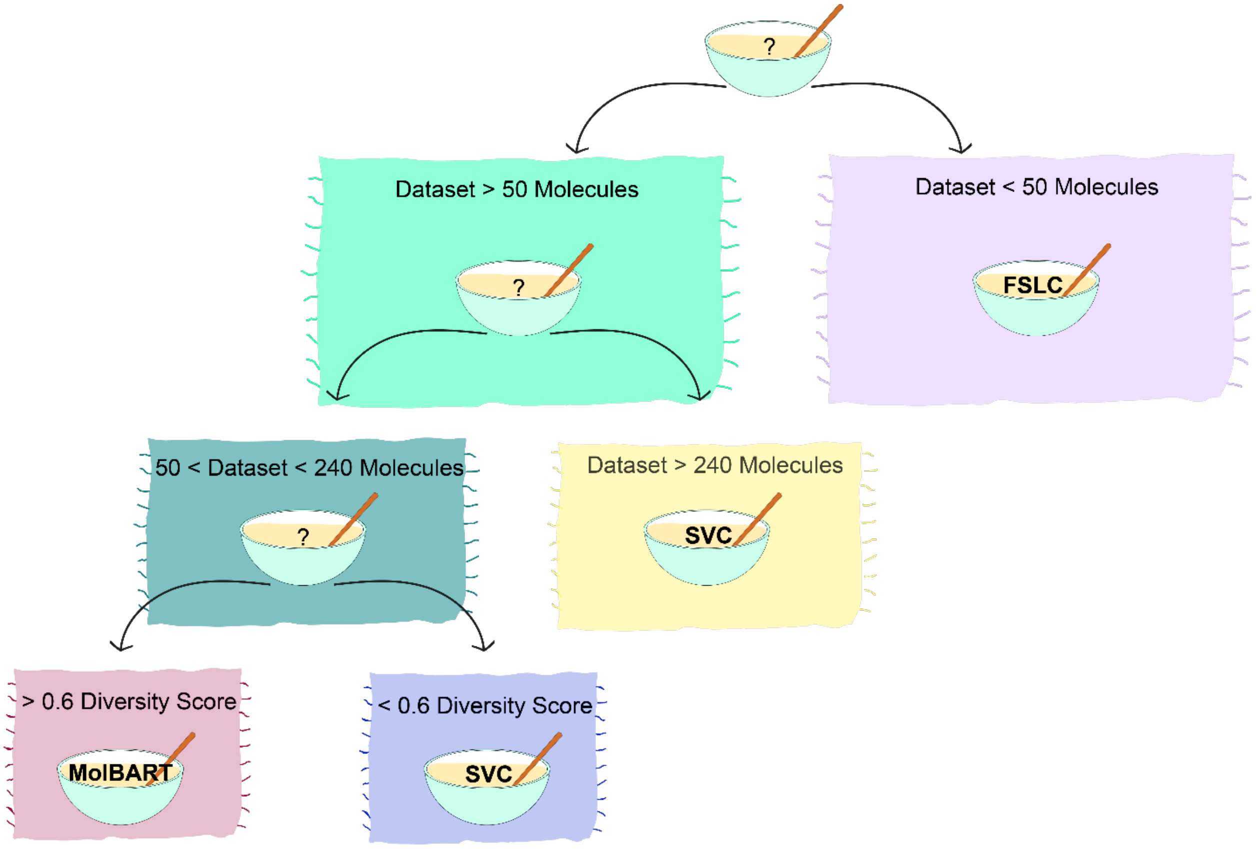

Figure S2. Stylized decision tree produced by the FIGS classifier model reveals a goldilocks zone paradigm for which ML model performs best based on dataset diversity and dataset size.

Supplementary Data

Supplementary Data 1. ChEMBL datasets and machine learning model R^2 comparison.
(See separate csv file)

Supplementary Tables

Table S1. The top 10 datasets with the largest differences between MolBART R^2 and SVR R^2

Test set	R2	R2	R2
size	MolBART	SVR	diff
16	0.04	-1.74	1.79
20	0.79	-0.41	1.19
32	0.91	-0.25	1.16
33	0.89	-0.24	1.13
34	0.93	-0.16	1.09
33	0.87	-0.22	1.09
32	0.84	-0.25	1.08
34	0.94	-0.12	1.05
25	0.83	-0.21	1.03
25	0.44	-0.59	1.03
27	0.86	-0.16	1.03

Table S2. 5-Fold Cross Validation Statistics for Traditional Machine Learning Algorithms.

GSK3β								
	AUC	F1 Score	Precision	Recall	Accuracy	Specificity	Cohen's Kappa	MCC
ADA	0.87	0.65	0.77	0.57	0.83	0.93	0.54	0.55
BNB	0.84	0.62	0.69	0.56	0.8	0.9	0.48	0.49
kNN	0.9	0.74	0.73	0.75	0.85	0.89	0.64	0.64
LREG	0.88	0.7	0.73	0.69	0.83	0.9	0.59	0.59
RF	0.91	0.73	0.79	0.69	0.86	0.93	0.64	0.64
SVC	0.91	0.74	0.7	0.79	0.84	0.86	0.63	0.63
XGB	0.9	0.74	0.76	0.73	0.86	0.91	0.64	0.64
ABL1								
	AUC	F1 Score	Precision	Recall	Accuracy	Specificity	Cohen's Kappa	MCC
ADA	0.95	0.89	0.92	0.86	0.89	0.93	0.79	0.79
BNB	0.92	0.85	0.88	0.83	0.86	0.9	0.73	0.73
kNN	0.95	0.88	0.87	0.89	0.88	0.88	0.77	0.77
LREG	0.95	0.89	0.9	0.89	0.9	0.9	0.79	0.79
RF	0.96	0.89	0.93	0.85	0.9	0.94	0.8	0.8
SVC	0.96	0.9	0.91	0.88	0.9	0.92	0.81	0.81
XGB	0.96	0.89	0.91	0.88	0.9	0.92	0.8	0.8
FYN								
	AUC	F1 Score	Precision	Recall	Accuracy	Specificity	Cohen's Kappa	MCC
ADA	0.82	0.43	0.84	0.33	0.93	0.99	0.41	0.47
BNB	0.87	0.57	0.76	0.46	0.94	0.99	0.54	0.56
kNN	0.85	0.7	0.78	0.65	0.95	0.98	0.68	0.68
LREG	0.83	0.63	0.8	0.53	0.95	0.99	0.6	0.62
RF	0.88	0.66	0.77	0.58	0.95	0.98	0.63	0.64
SVC	0.86	0.57	0.46	0.75	0.9	0.91	0.52	0.54
XGB	0.83	0.63	0.76	0.55	0.94	0.98	0.6	0.61
CDK5								
	AUC	F1 Score	Precision	Recall	Accuracy	Specificity	Cohen's Kappa	MCC
ADA	0.89	0.71	0.82	0.64	0.84	0.93	0.6	0.62
BNB	0.86	0.67	0.79	0.6	0.81	0.91	0.54	0.56
kNN	0.89	0.75	0.81	0.72	0.84	0.91	0.64	0.65
LREG	0.9	0.78	0.82	0.76	0.86	0.91	0.68	0.69
RF	0.9	0.7	0.82	0.63	0.83	0.92	0.58	0.6
SVC	0.87	0.77	0.79	0.77	0.85	0.89	0.66	0.67
XGB	0.89	0.74	0.85	0.67	0.85	0.94	0.64	0.65
MARK1								
	AUC	F1 Score	Precision	Recall	Accuracy	Specificity	Cohen's Kappa	MCC
ADA	0.9	0.2	0.2	0.2	0.73	0.93	0.13	0.13
BNB	0.7				0.67	0.93	-0.07	-0.07
kNN	0.6	0.2	0.2	0.2	0.67	0.83	0.03	0.03

LREG	0.73	0.2	0.2	0.2	0.73	0.93	0.13	0.13
RF	0.93	0.2	0.2	0.2	0.73	0.93	0.13	0.13
SVC	0.47	0.4	0.4	0.4	0.8	0.93	0.33	0.33
XGB	0.93	0.87	0.8	1	0.9	0.87	0.8	0.83

Table S3. External Test Set Statistics for Traditional Machine Learning Algorithms.

GSK3B	AUC	F1 Score	Precision	Recall	Accuracy	Specificity	Cohen's Kappa	MCC
ADA	0.71	0.36	0.6	0.26	0.66	0.9	0.18	0.21
BNB	0.65	0.2	0.41	0.14	0.61	0.89	0.02	0.03
kNN	0.8	0.65	0.55	0.78	0.69	0.63	0.38	0.4
LREG	0.71	0.52	0.54	0.49	0.66	0.76	0.25	0.25
RF	0.74	0.34	0.5	0.25	0.63	0.85	0.12	0.13
SVC	0.73	0.48	0.51	0.45	0.64	0.75	0.21	0.21
XGB	0.66	0.48	0.49	0.47	0.62	0.71	0.18	0.18
Consensus	0.7	0.39	0.54	0.31	0.65	0.85	0.17	0.18

ABL1	AUC	F1 Score	Precision	Recall	Accuracy	Specificity	Cohen's Kappa	MCC
ADA	0.79	0.65	0.72	0.59	0.75	0.86	0.46	0.46
BNB	0.84	0.64	0.73	0.57	0.75	0.87	0.46	0.47
kNN	0.84	0.72	0.66	0.78	0.76	0.75	0.51	0.52
LREG	0.82	0.7	0.74	0.65	0.78	0.86	0.52	0.53
RF	0.86	0.64	0.82	0.52	0.77	0.93	0.49	0.51
SVC	0.83	0.67	0.83	0.56	0.79	0.93	0.53	0.55
XGB	0.85	0.69	0.77	0.62	0.78	0.89	0.53	0.53
Consensus	0.82	0.68	0.81	0.58	0.79	0.91	0.52	0.54

FYN	AUC	F1 Score	Precision	Recall	Accuracy	Specificity	Cohen's Kappa	MCC
ADA	0.53	0.15	0.63	0.08	0.7	0.98	0.08	0.15
BNB	0.45	0.09	0.9	0.04	0.71	1	0.06	0.16
kNN	0.52	0.23	0.38	0.16	0.66	0.88	0.06	0.07
LREG	0.49	0.2	0.3	0.14	0.64	0.85	0	0
RF	0.57	0.14	0.67	0.08	0.71	0.98	0.08	0.15
SVC	0.47	0.37	0.28	0.52	0.45	0.41	-0.05	-0.06
XGB	0.52	0.2	0.41	0.13	0.68	0.92	0.06	0.07
Consensus	0.52	0.12	0.76	0.06	0.71	0.99	0.08	0.16

CDK5	AUC	F1 Score	Precision	Recall	Accuracy	Specificity	Cohen's Kappa	MCC
ADA	0.61	0.52	0.6	0.47	0.56	0.66	0.12	0.13
BNB	0.61	0.02	0.5	0.01	0.48	0.99	0	0
kNN	0.53	0.5	0.54	0.47	0.52	0.57	0.04	0.04
LREG	0.63	0.58	0.6	0.56	0.58	0.6	0.16	0.16
RF	0.64	0.51	0.83	0.37	0.63	0.92	0.28	0.34
SVC	0.62	0.57	0.61	0.53	0.58	0.64	0.17	0.17
XGB	0.58	0.56	0.58	0.55	0.56	0.58	0.12	0.12
Consensus	0.6	0.55	0.69	0.45	0.61	0.78	0.23	0.24

Table S4. Positively identified MARK1 Inhibitors from the initial screen were followed up with Dose-response curves. True MARK1 Inhbitors (see Figure 7 for experimental results) are labeled as Active=1. Predicted Actives by Few-Shot Learning model show high precision.

Name	Active (Measured)	Predicted (FSL)
ruboxistaurin HCl	0	0
fingolimod	0	0
Tofacitinib citrate	1	1
Crizotinib	0	0
Bosutinib	0	0
Ceritinib	0	0
AT9283	1	1
ON123300	1	0
baricitinib	1	1
Upadacitinib	1	1
Levobunolol (hydrochloride)	0	0
Closantel	0	0
Wedelolactone	0	0

Table S5. Most similar molecules in the MARK1 model.

query	query_name	MARK1 Closest Compound	target_name	Max Tanimoto
O=C(Nc1cn[nH]c1-c1nc2ccc(CN3CCOCC3)cc2[nH]1)NC1CC1	AT9283	Cc1ccc(-n2nc(C(C)(C)C)cc2NC(=O)Nc2ccc(OCCN3CCOCC3)c3ccccc23)cc1	MARK1	0.75
CCS(=O)(=O)N1CC(CC#N)(n2cc(-c3ncnc4[nH]ccc34)cn2)C1	baricitinib	CNC(=O)c1ccc(Nc2ncc(C(F)(F)F)c(NCc3nccnc3N(C)S(C)(=O)=O)n2)cc1	MARK1	0.56
COc1cc(Nc2c(C#N)cnc3cc(OCCCN4CCN(C)CC4)c(OC)cc23)c(Cl)cc1Cl	Bosutinib	Cc1ccc(-n2nc(C(C)(C)C)cc2NC(=O)Nc2ccc(OCCN3CCOCC3)c3ccccc23)cc1	MARK1	0.64
Cc1cc(Nc2ncc(Cl)c(Nc3ccccc3S(=O)(=O)C(C)C)n2)c(OC(C)C)cc1C1CCNCC1	Ceritinib	Cc1c(C(=O)N2CCOc3ccc(-c4ccc(N)nc4)cc3C2)ccc(S(C)(=O)=O)c1F	MARK1	0.66
Cc1cc(Nc2ncc(Cl)c(Nc3ccccc3S(=O)(=O)C(C)C)n2)c(OC(C)C)cc1C1CCNCC1	Ceritinib	Oc1cccc(Nc2ccnc3[nH]c4ccccc4c23)c1	MARK1	0.36
C[C@@H](Oc1cc(-c2cnn(C3CCNCC3)c2)cnc1N)c1c(Cl)ccc(F)c1Cl	Crizotinib	Cc1ccc(-n2nc(C(C)(C)C)cc2NC(=O)Nc2ccc(OCCN3CCOCC3)c3ccccc23)cc1	MARK1	0.62
CN1CCN(c2ccc(Nc3ncc4cc(C#N)c(=O)n(C5CCCC5)c4n3)cc2)CC1	ON123300	C[C@@H]1C[C@H]1C(=O)N1CCN(c2cnc(C#N)c(-c3cnn(C)c3)n2)C[C@H]1C	MARK1	0.73
C[C@@H]1CCN(C(=O)CC#N)C[C@@H]1N(C)c1ncnc2[nH]ccc12	Tofacitinib citrate	C[C@@H]1C[C@H]1C(=O)N1CCN(c2cnc(C#N)c(-c3cnn(C)c3)n2)C[C@H]1C	MARK1	0.79
CC[C@@H]1CN(C(=O)NCC(F)(F)F)C[C@@H]1c1cnc2cnc3[nH]ccc3n12	Upadacitinib	CC(=O)N1CC[C@@H](Nc2cncc(C(=O)c3cn(C(C)C)c4ncnc(N)c34)n2)[C@H]1c1ccc(F)cc1	MARK1	0.59

Supplemental references

Vella, D.; Ebejer, J.-P., Few-Shot Learning for Low-Data Drug Discovery. *Journal of Chemical Information and Modeling* **2023**, 63, 27-42.
