# Supplementary material for: The Goldilocks paradigm: comparing classical machine learning, large language models, and few-shot learning for drug discovery applications
Source: Commun Chem. 2024 Jun 12;7:134. doi: 10.1038/s42004-024-01220-4 (PMC11169557; doi:10.1038/s42004-024-01220-4)
Supplement: Supplementary file 2 — Description of Additional Supplementary Files [file 42004_2024_1220_MOESM2_ESM.pdf]

Description of Additional Supplementary Files

File name- Supplementary Data 1

File description- ChEMBL datasets and machine learning model R2 comparison.
